# Optimized Synthesis of New Thiosemicarbazide Derivatives with Tuberculostatic Activity

**DOI:** 10.3390/ijms222212139

**Published:** 2021-11-09

**Authors:** Corina Popovici, Cristina-Maria Pavel, Valeriu Sunel, Corina Cheptea, Dan Gheorghe Dimitriu, Dana Ortansa Dorohoi, Diana David, Valentina Closca, Marcel Popa

**Affiliations:** 1 Faculty of Chemistry, Alexandru Ioan Cuza University, 11 Carol I Blvd., 700506 Iasi, Romania; corinapopovici77@yahoo.com (C.P.); vsunel@uaic.ro (V.S.); 2 Department of Natural and Synthetic Polymers, Faculty of Chemical Engineering and Environmental Protection, “Gheorghe Sachi” Technical University, 73 Professor Dimitrie Mangeron Blvd., 700050 Iasi, Romania; marpopa@ch.tuiasi.ro; 3 Faculty of Physics, Alexandru Ioan Cuza University, 11 Carol I Blvd., 700506 Iasi, Romania; cristinamariadulgheriu@yahoo.com (C.-M.P.); dimitriu@uaic.ro (D.G.D.); ddorohoi@uaic.ro (D.O.D.); vclosca@gmail.com (V.C.); 4 Department of Biomedical Sciences, Faculty of Medical Bioengineering, “Grigore T. Popa” University of Medicine and Pharmacy, 9-13 M. Kogalniceanu Str., 700454 Iasi, Romania; 5 Warwick Medical School, University of Warwick, Coventry CV4 7AL, UK; diana.david@warwick.ac.uk; 6 Department of Science, “Eudoxiu Hurmuzachi” National College, 5 Calea Bucovinei Str., 725400 Radauti, Romania

**Keywords:** 2-mercapto-benzimidazole, sulfonic derivatives, heterocyclic thiosemicarbazides, tuberculostatic activity

## Abstract

Original results are presented in the field of research that addresses the extension of the reaction of residue of acyl-thiosemicarbazide fixation on the structure of 5-nitrobenzimidazole by a sulphonic group. The aim of the study is the increase of new thiosemicarbazide derivatives’ applicative potential in the field of biochemistry, with a wide range of medical applications. The newly obtained compounds were characterized by using elemental analysis and spectral analysis (FT-IR and ^1^H NMR). A study regarding the optimization of the chemical reactions was made. The performed in vitro biological tests confirmed the tuberculostatic activity of three newly obtained compounds against *Mycobacterium tuberculosis*.

## 1. Introduction

Studies in the literature discuss heterocyclic compounds at length, considering how they have contributed to the development of the society from biological point of view, as well as in terms of quality of life [1]. In their research, many scientists have developed an important variety of bioactive heterocyclic molecules. Between these, sulfur and nitrogen-containing compounds, such as thiosemicarbazides, have received significant attention, due to their tuberculostatic activity, in particular [2,3,4,5,6].

Although many compounds with antituberculosis activity are known, in modern chemotherapy the research, directions are oriented towards obtaining new compounds with delayed chemoresistance, non-toxicity, as well as to elucidate the mechanism of action on the Koch bacillus and to establish the links between chemical structure and chemotherapeutic activity.

Studying the correlation between the chemical structure and the biological action exerted on the Koch bacillus [7,8,9,10], it was considered that this is related to the properties that thiosemicarbazides form with certain metal ions, especially with copper ions, and some soluble metal complexes. By complexing copper ions, thiosemicarbazides block the bacterial enzymes, whose existence requires the presence of this element.

Other researchers [11,12,13,14,15,16] claim that an important role in the antibacterial activity of thiosemicarbazides belongs to the group 
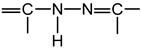
; indeed, acyl-thiosemicarbazides in their thiol form 
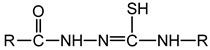
 contain this group.

The research highlights the importance of the nature and position of the radicals in the molecule, especially given the large number of compounds in which such dependencies between properties and substituents have been observed.

Thus, it is interesting to note its antibacterial [17,18,19,20,21], anticonvulsant [22], antifungal [23,24], cytostatic [25,26,27], and antioxidant [28,29] activity, especially when the thiosemicarbazide group is related to a heterocycle [15].

Based on the above, it can be concluded that an important role in the pharmacological activity of thiosemicarbazide derivatives has the structure of the molecule as a whole.

## 2. Results and Discussion

### 2.1. Synthesis of New Thiosemicarbazides

This paper presents the synthesis of new thiosemicarbazides whose active group has as support the rest of the ethyl ester of 5-nitrobenzimidazol-2-yl-sulfonyl-acetic acid.

**Scheme 1:**
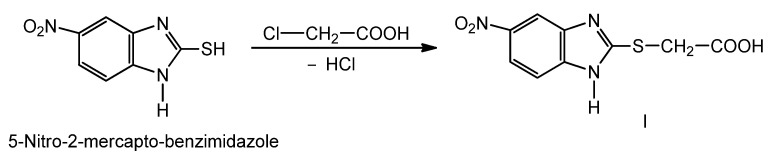
The synthesis of 5-nitro-benzimidazole-2-yl-mercapto-acetic acid (**I**).

A number of intermediates are required to obtain such combinations, such as 5-nitrobenzimidazol-2-yl-mercapto-acetic acid (**I**), which was obtained by reacting the 5-nitro-2-mercaptobenzimidazole with monochloroacetic acid in hot water (Scheme 1).

The compound was characterized in the terms of physical properties and the structure was confirmed by elemental and spectral analysis (FT-IR, ^1^H-NMR).

The IR spectra contain the band corresponding to the N–H vibration in the imidazole nucleus at 2929 cm^−1^, while the band specific to the C=N group appears at 1622 cm^−1^. The low frequency of this group is explained by its incorporation into the extended conjugate system between the two nitrogen atoms. The presence of the nitro group is confirmed by the symmetric and asymmetric vibrations at 1348 cm^−1^ and 1517 cm^−1^, respectively, in IR spectra. The C–S group is highlighted at 750 cm^−1^. The high intensity IR band at 3414 cm^−1^ corresponds to the COOH vibration.

The proton of the COOH group is highlighted at 11.58 ppm, while the proton of the N–H group appears in ^1^H-NMR spectra at 6.12–6.14 ppm. Aromatic protons are found in the range 7.60–8.02 ppm.

**Scheme 2:**
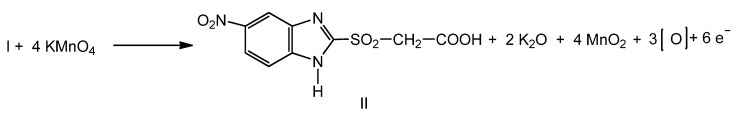
Synthesis of 5-nitrobenzimidazole-2-yl-sulphonyl-acetic acid (**II**).

The compound **I** was oxidized with a dilute aqueous solution of potassium permanganate, by heating in a water bath and thus it was possible to obtain the 5-nitrobenzimidazol-2-yl-sulfonyl-acetic acid (**II**) (Scheme 2).

A slight excess of potassium permanganate compared to the amount resulting from the stoichiometric calculation was used in the synthesis.

The compound was characterized in terms of the physical properties and structurally by elemental and spectral analysis (FT-IR, ^1^H-NMR).

In the IR spectra, the N–H group produces an average band at 2850 cm^−1^ and the C=N group gives a bit intense at 1681 cm^−1^. The symmetric and asymmetric NO_2_ vibration bands are found at 1342 cm^−1^ and 1516 cm^−1^, respectively. The absorption corresponding to the vibration of the C–S bond generates a band at the frequency of 752 cm^−1^, while the SO_2_ group has a specific band at 1495 cm^−1^. In the high frequency range, the peak characteristic of the COOH group appears at 3000 cm^−1^.

In the ^1^H-NMR spectra, the protons of the aromatic system were highlighted at 7.89–8.85 ppm, the proton of the N–H group at 5.40 ppm, the protons of the CH_2_ group at 3.36 ppm and the proton of the COOH group appears at 11.39–11.40 ppm.

**Scheme 3:**
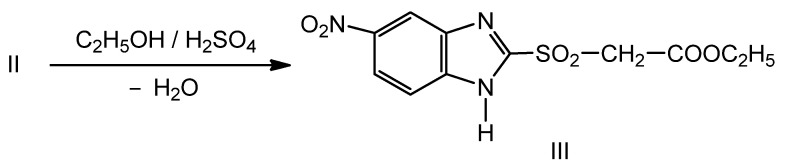
Synthesis of 5-nitrobenzimidazole-2-yl-sulphonyl-acetic acid ethyl ester (**III**).

The 5-nitrobenzimidazol-2-yl-sulphonyl-acetic acid (**II**), previously obtained by treatment with the anhydrous ethyl alcohol and in the presence of concentrated sulfuric acid, at reflux, was converted to its corresponding ethyl ester, **III** (Scheme 3).

After establishing some physical properties, the structure of the compound was determined by elemental and spectral analysis (FT-IR, ^1^H-NMR).

In the IR spectra, the N–H group is clearly identified by the band generated at 2947 cm^−1^. To this, the absorption of the valence vibration C=N at a frequency of 1623 cm^−1^ is added. The symmetric NO_2_ group gives intense absorption at 1337 cm^−1^ and the asymmetric NO_2_ group gives intense absorption at 1518 cm^−1^. The C–S and SO_2_–CH_2_ bonds produce absorptions at 749 cm^−1^ and 1493 cm^−1^, respectively.

Corresponding to the IR absorption of the ester group C=O, there is a single band of medium intensity at 1737 cm^−1^.

The value of the chemical shifts and the intensity of the peaks in the ^1^H-NMR spectra are in full agreement with the type and the number of protons in the compound **III**. The nitrogen proton at position 1 of the imidazole heterocycle corresponds to a singlet at δ = 5.52 ppm.

In the aliphatic zone, the protons of the methyl group are identified at δ = 1.18–1.22 ppm. At δ = 3.37 ppm, the protons of the CH_2_ group from –COOCH_2_–CH_3_ are also identified. The signal of the protons of the CH_2_ group, related to the sulfur from position 2 of the benzimidazole heterocycle, appears at 4.13–4.18 ppm and that of the aromatic protons appears at 7.87–8.84 ppm.

**Scheme 4:**
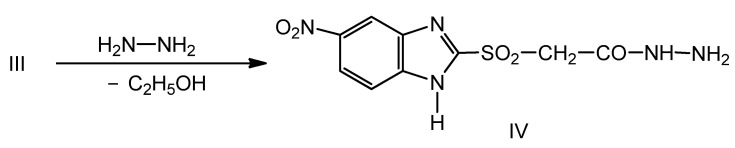
The synthesis of 5-nitrobenzimidazole-2-yl-sulphonyl-acetic acid hydrazide (**IV**).

The 5-nitrobenzimidazole-2-yl-sulphonyl-acetic acid ethyl ester **III** served as a precursor in the next step for obtaining 5-nitrobenzimidazole-2-yl-sulphonyl-acetic acid hydrazide **IV** (Scheme 4).

This hydrazide is obtained by the general method [30,31,32], a classic method which consists of treating the corresponding ester with hydrazine hydrate in an anhydrous ethyl alcohol medium, under reflux.

The structure of the compound, after establishing some physical properties, was confirmed by elemental and spectral analysis (FT-IT, ^1^H-NMR).

From the IR spectra of compound **IV**, it is found that the frequency of the valence vibration for the group C=O (hydrazide) is at 1659 cm^−1^ and the valence vibration of the N–H bonds appears at 3252 cm^−1^ and 3309 cm^−1^.

The two bands corresponding to the symmetric and asymmetric vibrations of the NO_2_ group were also identified at 1339 cm^−1^ and 1518 cm^−1^, respectively. At 790 cm^−1^ the spectra show the band characteristic of the C–S bond, and in the IR spectra, at 1494 cm^−1^ the band specific to the SO_2_ group appears.

The ^1^H-NMR spectra contain the NH_2_ proton signal at 4.22–4.34 ppm (d, 2H), the nitrogen proton at position 1 at 5.12–5.14 ppm, the nitrogen proton from–CO–NH–group at 9.65 ppm and the aromatic proton at 7.74–8.83 ppm.

The 5-nitrobenzimidazole-2-yl-sulphonyl-acetic acid hydrazide **IV** having the acyl-hydrazine group with the mobile hydrogen gave us the possibility to obtain, by treatment with aromatic isothiocyanates, reactive in turn due to the unsaturated group –N=C=S, compounds containing grafted on the residue 2-sulphonyl-5-nitrobenzimidazole acyl-thiosemicarbazide group.

**Scheme 5:**
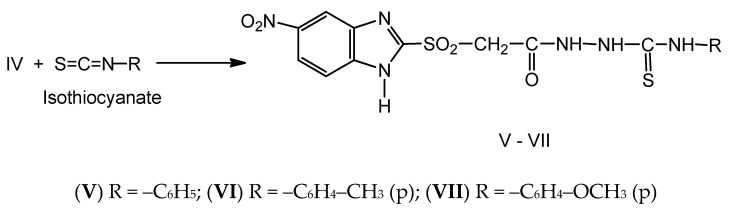
Synthesis of 1-(5′-nitrobenzimidazole-2′-yl-sulphonyl-acetyl)-4-aryl-thiosemicarbazides (**V**–**VII**).

The synthesis of 1-(5′-nitrobenzimidazole-2′-yl-sulphonyl-acetyl)-4-aryl-thiosemicarbazides was performed according to the procedures described in the literature for other types of thiosemicarbazides [2,6,33,34]: in methyl alcohol solution by heating to boiling a mixture of 5-nitrobenzimidazole-2-yl-sulphonyl-acetic acid hydrazide **IV** with isothiocyanates of: phenyl, para-tolyl, para-methoxyphenyl, (Scheme 5).

The thiosemicarbazides **V**–**VII** were characterized by some physical properties and their structures were confirmed by elemental analysis and FT-IR, ^1^H-NMR spectra.

**Figure 1:**
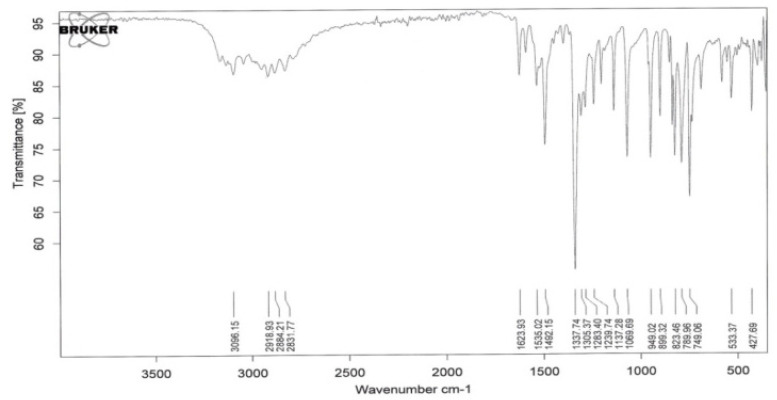
The FT-IR spectra of 1-(5′-nitrobenzimidazole-2′-yl-sulphonyl-acetyl)-4-(p-methoxyphenyl)-thiosemicarbazide (**VII**).

In the FT-IR spectra of the compounds **V**–**VII** (see Figure 1), in the area 2881–3098 cm^−1^ there are bands corresponding to the valence vibration of the associated N–H group. The nitro group gives the two bands generated by the vibrations of symmetric valence between 133 and 342 cm^−1^ and asymmetric between 152 and 535 cm^−1^, while at 749–752 cm^−1^ the band characteristic of the C–S connection is noticeable. The band from 1492 to 1498 cm^−1^ corresponds to the vibration of the SO_2_–CH_2_ connection. The C=O bond produces an average band between 162 and 629 cm^−1^ and the C=S group appears at 1137–1239 cm^−1^. The 746 cm^−1^, 758 cm^−1^ and 789 cm^−1^ IR bands are assigned to the monosubstituted and disubstituted benzene nucleus.

**Figure 2:**
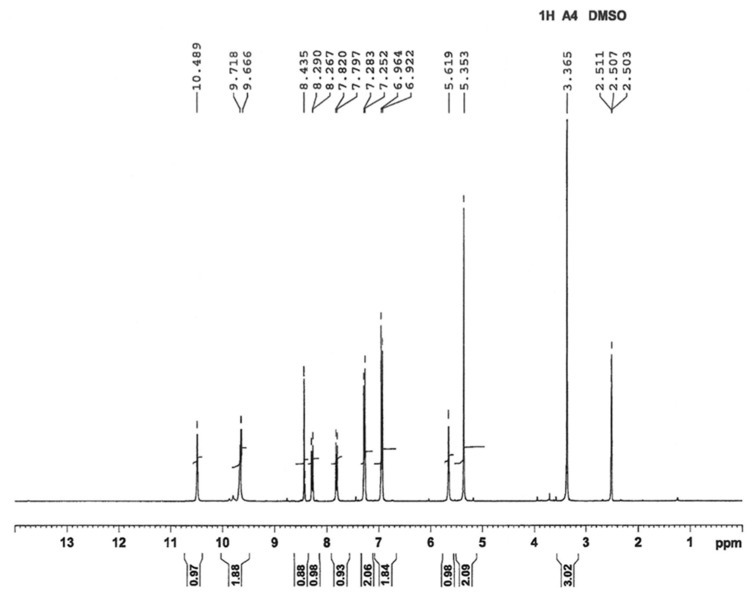
^1^H-NMR spectra of 1-(5′-nitrobenzimidazole-2′-yl-sulphonyl-acetyl)-4-(p-methoxyphenyl)-thiosemicarbazide (**VII**).

The ^1^H-NMR spectra show the presence of structural elements in each thiosemicarbazide (Figure 2). They contain the N–H proton signal from the benzimidazole heterocycle at 5.61–5.89 ppm (s, 1H). The protons of the methyl group at δ = 2.34 ppm for compound VI and δ = 3.36 ppm for the thiosemicarbazide **VII** are identified. The three methylene protons appear as a singlet at δ = 5.35–5.39 ppm and at δ = 9.66–9.89 ppm (d, 2H). At δ = 10.48–10.62 ppm (s, 1H) the protons bound to the nitrogen atom in the thiosemicarbazide chain appear. The aromatic protons in the phenyl ring and in the benzimidazole system were differentiated according to the neighborhoods and couplings at δ = 6.92–7.82 ppm and at 8.02–8.43 ppm.

### 2.2. Optimization of the Chemical Reactions for Obtaining the Thiosemicarbazides **V**–**VII**

The data (the dimensionless and the real (between parentheses) variables as well as the measured yield in each experiment) used in 3^2^ experiments [35,36,37], are organized in order to optimize the reaction’s yield, as shown in Table 1, Table 2 and Table 3.

By using data obtained in 3^2^ experiments and in the experiment organized in the center of the variable’s domain, the following equations were obtained:

(1) $$\eta =79.88+0.83x_{1}-0.67x_{2}-0.25x_{1}x_{2}-3.83x_{1}^{2}-3.67x_{2}^{2},\;\mathrm{for}\;\mathrm{the}\;\mathrm{compound}\;V$$

(2) $$\eta =84.33+0.33x_{1}-0.67x_{2}+0.25x_{1}x_{2}-3.00x_{1}^{2}-3.00x_{2}^{2},\;\mathrm{for}\;\mathrm{the}\;\mathrm{compound}\;\mathrm{VI}$$

(3) $$\eta =89.22-0.17x_{1}-0.50x_{2}-1.83x_{1}^{2}-3.84x_{2}^{2},\;\mathrm{for}\;\mathrm{the}\;\mathrm{compound}\;\mathrm{VII}$$

The dimensionless variables vary between −1 and +1.

**Figure 3:**
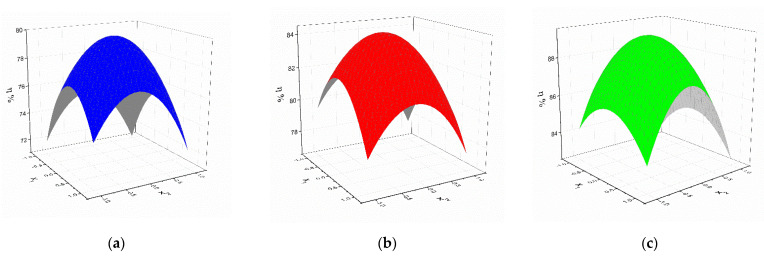
Dependence of the reaction’s yield on the dimensionless variables in optimization process of reactions from Scheme 5. for the compounds: (**a**) **V**; (**b**) **VI**; (**c**) **VII**.

The dependence of the reaction’s yield on the dimensionless variables is illustrated in Figure 3 for the three new compounds (**V**, **VI** and **VII**, respectively).

Using the relations (1–3), one can compute the favorable conditions for the chemical reactions in which the substances **V**–**VII** are obtained, in order to save precursor substances, energy and time. The most favorable conditions in which the obtaining reactions for compounds **V**–**VII** can take place are given in Table 4.

### 2.3. Study of Tuberculostatic Activity of Thiosemicarbazides **V**–**VII**

Knowing the importance of the thiosemicarbazide component in terms of the tuberculostatic activity of some compounds, we set out to test thiosemicarbazides **V**–**VII** from 5-nitrobenzimidazole-2-yl-sulphonyl-acetic acid hydrazide **IV** in terms of tuberculostatic activity, taking the isoniazid, a tuberculostatic drug in the clinical circuit, as a reference.

The tests were performed on *Mycobacterium tuberculosis*, applying the method of serial dilution, on liquid medium, using Youmans medium with bovine serum. The *Mycobacterium tuberculosis* strain was inoculated at a concentration of 0.01 mg/5 mL culture medium. For testing, the solutions of thiosemicarbazides **V**–**VII** in DMSO were obtained by dissolving 100 μg thiosemicarbazide in a mixture of dimethyl sulfoxide and phosphate buffer (pH = 7), in a volumetric ratio of 1:4 (*V*/*V*). The concentration of the thiosemicarbazides in the culture medium was varied (5, 10, 20, 30, 40 μg/mL) and it was found that at low concentrations (below 10 μg/mL) clear determinations of tuberculostatic activity cannot be made.

The readings were performed at 6 and 15 days after inoculation, respectively; the results are presented in Table 5.

The tests performed on *Mycobacterium tuberculosis*, based on isonicotinic acid hydrazide (IAH), show that at concentrations of at least 30 μg/mL, the thiosemicarbazides **V**–**VII** show moderate tuberculostatic activity. The most active in inhibiting the development of the tuberculosis bacillus are the compounds **VI** and **VII**, at concentrations of at least 20 μg/mL.

At values below 10 μg/mL, no tested compound shows tuberculostatic activity. Of the thiosemicarbazides **V**–**VII**, the compound **VII** is the most active against *Mycobacterium tuberculosis*, probably due to the existence of the methoxy group on the benzene nucleus of the thiosemicarbazide residue.

Comparing the values of the minimum inhibitory concentration (MIC), it is observed that all the thiosemicarbazides tested have the minimum inhibitory concentration higher than the isonicotinic acid hydrazide. The compound **VII** has the closest MIC value to that of the reference tuberculostatic drug. However, the tuberculostatic activity of the compounds **V**–**VII** was demonstrated and future studies could lead to the improvement of their properties in order to be used as drugs against *Mycobacterium tuberculosis*. Moreover, the structure 2-mercapto-benzimidazole allows the effective functionalization of SH group, subsequently favoring the introduction of bioactive molecular segments, with the benzimidazole itself having the role of carrier to the target cells.

## 3. Materials and Methods

### 3.1. Chemical Compounds and Analysis

The chemical reactants were purchased from Merck and Fluka (now Merck) companies and used without any purification.

The purity of the obtained compounds was checked by quantitative elemental analysis, Fourier-transform infrared (FT-IR) spectroscopy, and nuclear magnetic resonance (NMR) spectroscopy.

Quantitative elemental analysis was performed by using the Exeter Analytical CE440.

The FT-IR spectra of all compounds were recorded by using a BRUKER Tensor-27 FT-IR (ATR) spectrophotometer.

BRUKER ARX 400 spectrometer equipped with 5 mm QNP ^1^H/^13^C/^31^P/^19^F samples and Silicon Graphics INDIGO^2^ workstation was used to record the ^1^H NMR spectra (DMSO-d_6_, 400 MHz) of the new compounds.

The obtained results of these analyses are detailed below.

**5-Nitrobenzimidazole-2-yl-mercapto-acetic acid (I).**

To give a clear solution, 18.9 g (0.2 mol) Monochloroacetic acid is dissolved in 300 mL of distilled water; 39 g (0.2 mol) of 5-nitro-2-mercapto-benzimidazole was added in portions, vigorously stirred and the solution was heated to boiling for 3 h. The liquid is filtered hot, and, after cooling, the 5-nitrobenzimidazole-2-yl-mercapto-acetic acid was separated in the form of an abundant precipitate. The compound was filtered by vacuum, dried and purified by recrystallization from boiling water.

White-yellow solid (49.9 g; yield 89%). M.p. = 208–210 °C.

Elemental analysis: Calculated for C_9_H_7_N_3_O_4_S (%): C, 42.68; H, 2.76; N, 16.60; S, 12.64. Found (%): C, 42.80; H, 2.98; N, 16.91; S, 12.83.

FT-IR, γ_max_ cm^−1^: 2929 (NH); 1622 (C=N); 1346 (NO_2_ symm); 1547 (NO_2_ assym); 750 (C–S); 809 (S–CH_2_); 3414 (COOH).

^1^H-NMR (DMSO-d_6_, 400 MHz), δ ppm: 3.37 (s, 2H, CH_2_); 6.12–6.14 (s, 1H, NH); 7.60–7.68 (d, 1H, CHAr); 8.00–8.02 (d, 1H, CHAr); 11.58 (d, 1H, COOH).

**5-Nitrobenzimidazole-2-yl-sulphonyl-acetic acid (II).**

Then, 7.59 g (0.03 mol) of 5-Nitrobenzimidazole-2-yl-mercapto-acetic acid (**I**) was suspended in 150 mL of distilled water. The reaction mixture was heated in a water bath and then 6.39 g (0.04 mol) of fine powdered potassium permanganate was added in small portions under continuous stirring for 60 min. The manganese dioxide separates as brown precipitate.

The reaction continues by heating for another 40–50 min. The solution is discolored, and the manganese dioxide is deposited on the bottom of the reaction flask. After filtration, the potassium salt solution of the 5-nitrobenzimidazole-2-yl-sulphonyl-acetic acid is concentrated to 1/4 of the initial volume. By acidification with hydrochloric acid diluted to pH = 3.5–4, under stirring and cooling, the corresponding acid **II** separates. The crude product is purified by recrystallization from anhydrous ethyl alcohol.

Light yellow solid (6.32 g; yield 74%). M.p. = 135–136 °C.

Elemental analysis: Calculated for C_9_H_7_N_3_O_6_S (%): C, 37.89; H, 2.45; N, 14.73; S, 11.22. Found (%): C, 38.13; H, 2.64; N, 15.06; S, 11.48.

FT-IR; γ_max_ cm^−1^: 2850 (NH); 1681 (C=N); 1342 (NO_2_ symm); 1516 (NO_2_ assym); 752 (C–S); 1495 (SO_2_–CH_2_); 3000 (COOH).

^1^H-NMR (DMSO-d_6_, 400 MHz), δ ppm: 3.36 (s, 2H, CH_2_); 5.41 (s, 1H, NH); 7.89–7.92 (d, 1H, CHAr); 8.07–8.09 (d, 1H, CHAr); 8.85 (s, 1H, CHAr); 11.39–11.40 (s, 1H, COOH).

**5-Nitrobenzimidazole-2-yl-sulphonyl-acetic acid (III) ethyl ester.**

1.95 g (0.007 mol) of 5-nitrobenzimidazole-2-yl-sulphonyl-acetic acid (**II**) are placed in a flask equipped with a cold water jacket, to which 250 mL of anhydrous ethyl alcohol and 2 mL of concentrated sulfuric acid are added. The contents are refluxed for 3 h, after which the excess of alcohol is removed by distillation under reduced pressure. The concentrated solution is treated with 100 mL of distilled water and then brought to pH = 7 by treatment with sodium carbonate. The ester is extracted into ethyl ether and, after removal under vacuum, a glue-like product results which, by repeated washing with anhydrous ethyl ether, solidifies. The compound is purified by recrystallization from anhydrous ethyl alcohol.

Yellow solid (1.60 g; yield 76%). M.p. = 110–112 °C

Elemental analysis: Calculated for C_11_H_11_N_3_O_6_S (%): C, 43.13; H, 3.51; N, 13.41; S, 10.22. Found (%): C, 43.35; H, 3.69; N, 13.72; S, 10.59.

FT-IR; γ_max_ cm^−1^: 2947 (NH); 1623 (C=N); 1337 (NO_2_ symm); 1518 (NO_2_ assym); 749 (C–S); 1493 (SO_2_–CH_2_); 1737 (CO ester).

^1^H-NMR (DMSO-d_6_, 400 MHz), δ ppm: 1.18–1.22 (t, 3H, CH_3_); 3.37 (s, 2H, CH_2_); 4.13–4.18 (c, 2H, CH_2_); 5.52 (s, 1H, NH); 7.87–7.90 (d, 1H, CHAr); 8.24–8.27 (d, 1H, CHAr); 8.84 (s, 1H, CHAr).

**5-Nitrobenzimidazole-2-yl-sulphonyl-acetic acid (IV) hydrazide.**

Here, 6.26 g (0.02 mol) of 5-nitrobenzimidazol-2-yl-sulphonyl-acetic acid ethyl ester (**III**) is dissolved in 30 mL of anhydrous ethyl alcohol by gentle heating; 4 mL of 98% hydrazine hydrate are added and then one reflux for 3 h.

After partial removal of the solvent, on cooling, the hydrazide is separated. The hydrazide is filtered, dried in a vacuum oven, and purified by recrystallization from ethyl alcohol in boiling process.

White-yellow solid (4.86 g; yield 82%). M.p. = 226–228 °C

Elemental analysis: Calculated for C_9_H_9_N_5_O_6_S (%): C, 36.12; H, 3.01; N, 23.41; S, 10.70. Found (%): C, 36.35; H, 3.37; N, 23.58; S, 10.99.

FT-IR; γ_max_ cm^−1^: 3252, 3309 (NH); 1614 (C=N); 1339 (NO_2_ symm); 1518 (NO_2_ assym); 790 (C–S); 1494 (SO_2_–CH_2_); 1659 (C=O hydrazide).

^1^H-NMR (DMSO-d_6_, 400 MHz), δ ppm: 3.79 (s, 2H, CH_2_); 4.22–4.34 (d, 2H, CH_2_); 5.12–5.14 (s, 1H, NH); 7.74–7.76 (d, 1H, CHAr); 8.19–8.27 (d, 1H, CHAr); 8.83 (s, 1H, CHAr); 9.65 (d, 1H, NH).

**1-(5′-Nitrobenzimidazole-2′-yl-sulphonyl-acetyl)-4-aryl-thiosemicarbazide (V–VII).**

Here, 0.05 mol 5-Nitrobenzimidazole-2-yl-sulphonyl-acetic acid hydrazide (IV) is dissolved in 75 mL of anhydrous methyl alcohol by gentle heating. Then, 0.005 moles of aromatic isothiocyanate in 10 mL of anhydrous methyl alcohol is added to the obtained solution. The reaction mixture is refluxed in a water bath for 3–4 h.

A crystalline precipitate appears after 45–50 min of heating, which becomes more and more abundant during heating. The precipitate is cooled, filtered in vacuum and dried. The crude product is purified by recrystallization from methyl alcohol.

**1-(5′-Nitrobenzimidazole-2′-yl-sulphonyl-acetyl)-4-phenyl-thiosemicarbazide (V).**

Light yellow solid (1.70 g; yield 79%). M.p. = 240–242 °C.

Elemental analysis: Calculated for C_16_H_14_N_6_O_5_S_2_ (%): C, 46.23; H, 3.22; N, 19.35; S, 14.76. Found (%): C, 44.42; H, 3.43; N, 19.67; S, 15.11.

FT-IR; γ_max_ cm^−1^: 2918, 2948, 3096 (NH); 1612 (C=N); 1338 (NO_2_ symm); 1535 (NO_2_ assym); 750 (C–S); 1492 (SO_2_–CH_2_); 1623 (C=O); 1137 (C=S); 746 (monosubstituted benzene nucleus).

^1^H-NMR (DMSO-d_6_, 400 MHz), δ ppm: 5.39 (s, 2H, CH_2_); 5.71 (s, 1H, NH); 7.16–7.19 (d, 1H, CHAr); 7.33–7.41 (m, 4H, CHAr); 7.69–7.74 (d, 1H, CHAr); 8.03–8.06 (d, 1H, CHAr); 8.21 (s, 1H, CHAr); 9.79–9.81 (d, 2H, NH); 10.62 (s, 1H, NH).

**1-(5′-Nitrobenzimidazole-2′-yl-sulphonyl-acetyl)-4-(p-tolyl)-thiosemicarbazide (VI).**

Yellow solid (1.85 g; yield 83%). M.p. = 246–247 °C.

Elemental analysis: Calculated for C_17_H_16_N_6_O_5_S_2_ (%): C, 45.53; H, 3.57; N, 18.75; S, 14.67. Found (%): C, 45.72; H, 3.73; N, 19.09; S, 14.67.

FT-IR; γ_max_ cm^−1^: 2881, 2940, 3098 (NH); 1615 (C=N); 1342 (NO_2_ symm); 1525 (NO_2_ assym); 752 (C–S); 1498 (SO_2_–CH_2_); 1629 (C=O); 1224 (C=S); 758 (para-disubstituted benzene nucleus).

^1^H-NMR (DMSO-d_6_, 400 MHz), δ ppm: 2.34 (s, 3H, CH_3_); 5.38 (s, 2H, CH_2_); 5.89 (s, 1H, NH); 7.19–7.21 (d, 1H, CHAr); 7.28–7.31 (d, 2H, CHAr); 8.28–8.29 (d, 1H, CHAr); 8.32 (s, 1H, CHAr); 9.77–9.83 (d, 2H, NH); 10.54 (s, 1H, NH).

**1-(5′-Nitrobenzimidazol-2′-yl-sulphonyl-acetyl)-4-(p-methoxy phenyl)-thiosemicarbazide (VII).**

Light yellow solid (2.03 g; yield 88%). M.p. = 249–251 °C.

Elemental analysis: Calculated for C_17_H_16_N_6_O_6_S_2_ (%): C, 43.96; H, 3.44; N, 18.10; S, 13.79. Found (%): C, 44.11; H, 3.71; N, 18.42; S, 14.08.

FT-IR; γ_max_ cm^−1^: 2884, 2918, 3096 (NH); 1337 (NO_2_ symm); 1535 (NO_2_ assym); 749 (C–S); 1492 (SO_2_–CH_2_); 1623 (C=O); 1239 (C=S); 789 (para-disubstituted benzene nucleus).

^1^H-NMR (DMSO-d_6_, 400 MHz), δ ppm: 3.36 (s, 3H, CH_3_); 5.35 (s, 2H, CH_2_); 5.61 (s, 1H, NH); 6.92–6.96 (d, 2H, CHAr); 7.25–7.28 (d, 2H, CHAr); 7.79–7.82 (d, 1H, CHAr); 8.26–8.29 (d, 1H, CHAr); 8.43 (s, 1H, CHAr); 9.66–9.71 (d, 2H, NH); 10.48 (s, 1H, NH).

### 3.2. Optimization of the Chemical Reactions

In order to establish the most favorable conditions for chemical reactions, factorial experiments were made, taking the reaction yield as an indicator of the reaction optimization. The reaction yield significantly depends on the real variables temperature and time of reaction; *X*_1_ and *X*_2_ are considered as the real variables in the experiment.

Instead of real variables, dimensionless variables *x*_1_ and *x*_2_ are considered, with variation domains between −1 and +1.

If one defines $\bar{X_{i}}$ the average value of the real variables, with extreme values *X_im_* and *X_iM_*, *i* = 1, 2, with indices *m* and *M* for minimum and maximum values, respectively,

(4) $$\bar{X_{i}}=\frac{X_{im}+X_{iM}}{2},\;i=1,2,$$

and Δ*X_i_* the half of the variation domain of the variables

(5) $$\Delta X_{i}=\frac{X_{iM}-X_{im}}{2},\;i=1,2,$$

the dimensionless variables are defined by:

(6) $$x_{i}=\frac{X_{i}-\bar{X_{i}}}{\Delta X_{i}},\;i=1,2.$$

The variation domain of the dimensionless variables is −1 to 1, as it results from their minimum and maximum values:

(7) $$x_{im}=\frac{X_{im}-\bar{X_{i}}}{\Delta X_{i}}=-1,\;i=1,2,$$

(8) $$x_{iM}=\frac{X_{iM}-\bar{X_{i}}}{\Delta X_{i}}=1,\;i=1,2.$$

Let us suppose that the reaction yield, *η*, depends on the individual influence of *x*_1_ and *x*_2_, as well as on the conjugate effects of the dimensionless variables, as relation (9) shows:

(9) $$\eta =a_{0}+a_{1}x_{1}+a_{2}x_{2}+a_{12}x_{1}x_{2}+a_{11}x_{1}^{2}+a_{22}x_{2}^{2}.$$

In relation (9), the last three terms express the conjugate effects of the dimensionless variables. The absolute values of the coefficients *a*_1_, *a*_2_, *a*_11_, *a*_12_ and *a*_22_ indicate the strength of the reaction yield dependence on the dimensionless variables. Their positive sign shows the increase in the reaction yield with the variable increasing, while the negative sign indicates the decrease in the reaction yield when the variable increases.

If the reaction yield depends only on two variables, the coefficients *a*_1_, *a*_2_, *a*_11_, *a*_12_, *a*_22_ can be estimated by measuring the reaction yield in a 3^2^ factorial experiment. The regression coefficients *a*_1_, *a*_2_, *a*_11_, *a*_12_ and *a*_22_ are determined using the values of the reaction yield *η* measured for nine values of the two dimensionless variables, at the extremes and in the middle of their variation domain. The dimensionless variables must satisfy the principle of orthogonality. For this reason, $x_{1}^{2}$ and $x_{2}^{2}$ are changed in $x_{1}^{2}-2/3$ and $x_{2}^{2}-2/3$, respectively (see also Table 1, Table 2 and Table 3). In this case, one obtains:

(10) $$\eta =a_{0}+a_{1}x_{1}+a_{2}x_{2}+a_{12}x_{1}x_{2}+a_{11}\left(x_{1}^{2}-2/3\right)+a_{22}\left(x_{2}^{2}-2/3\right).$$

The equations used to determine the regression coefficients are obtained by solving the system with experimental data and taking into consideration the orthogonality of the dimensionless variables:

(11) $$a_{0}=\bar{\eta }-2/3\left(a_{11}+a_{22}\right),$$

(12) $$\bar{\eta }=1/9\sum_{k=1}^{9}\eta_{k},$$

(13) $$a_{i}=\frac{\sum_{k}\eta_{k}x_{ik}}{\sum_{k}x_{ik}^{2}},\;i=1,2,$$

(14) $$a_{12}=\frac{\sum_{k}\eta_{k}x_{1k}x_{2k}}{\sum_{k}x_{1k}^{2}x_{2k}^{2}},$$

(15) $$a_{ii}=\frac{\sum_{k}\eta_{k}\left(x_{ik}^{2}-2/3\right)}{\sum_{k}{\left(x_{ik}^{2}-2/3\right)}^{2}},\;i=1,2.$$

In order to check if the relations (1–3) correctly describe the effects of the dimensionless variables, an additional experiment must be organized in the middle of the variation domain of these variables, by measuring the reaction yield in this point. The experiment is repeated three times. One obtains the values *η*_10_, *η*_20_, *η*_30_ and the average value $\bar{\eta }=1/3\left(\eta_{10}+\eta_{20}+\eta_{30}\right)$. One calculates the square of average error

(16) $$S_{\eta }^{2}=\frac{\sum_{i=1}^{3}{\left(\bar{\eta_{0}}-\eta_{i0}\right)}^{2}}{n-1}$$

and the precision of the measurements

(17) $$P=\sqrt{\frac{S_{\eta }^{2}}{N}},$$

where *N* is the number of the measurements in the 3^2^ factorial experiment.

If one considers that each regression coefficient is estimated with the same precision *P*, one can compute Student’s *t*-test [38,39] for each coefficient:

(18) $$t_{j}=\frac{\left|a_{j}\right|}{P},\;\mathrm{or}\;t_{ij}=\frac{\left|a_{ij}\right|}{P},\;i,j=1,2.$$

Leave the values of *t_j_* smaller than 3 for which the corresponding regression coefficient does not influence the reaction yield. All regression coefficients with a Student’s *t*-test value of higher than 3 have significant importance for the reaction yield.

### 3.3. Biological Tests

The method of serial dilution using Youman’s medium with bovine serum was applied on *Mycobacterium tuberculosis* to test the tuberculostatic activity of the thiosemicarbazides **V**–**VII**. Isoniazid was used as a reference drug.

## 4. Conclusions

In order to find active tuberculostatic compounds against *Mycobacterium tuberculosis* and to establish structure-activity relationships in the series of synthesized heterocyclic thiosemicarbazides, seven new compounds were synthetized that are not described in the literature.

The 2-sulphonyl-5-nitrobenzimidazole molecule was used to achieve a selective transporter of groups with tuberculostatic properties in the body or in the target tissues.

Methods of selective sulfonation of 5-nitrobenzimidazole-2-yl-mercapto-acetic acid and of forming 5-nitrobenzimidazole-2-yl-sulphonyl-acetic acid ester have been developed.

In this study, 5-Nitrobenzimidazole-2-yl-sulphonyl-acetic acid hydrazide was prepared, and, by its addition to some aromatic isothiocyanates, the corresponding series of heterocyclic acyl-sulphonyl-thiosemicarbazides was obtained.

The synthesized intermediates and final products were characterized using elemental and spectral analysis (FT-IR, ^1^H-NMR).

The reactions for obtaining the new thiosemicarbazides were optimized by statistical models based on the factorial design.

Some aspects regarding the tuberculostatic action of the synthesized thiosemicarbazides were elucidated through the performed biological tests.

## Figures and Tables

**Table 1:** Dimensionless variables and the measured reaction’s yield for obtaining the compound **V**.

No.	*x*_1_; *T* (°C)	*x*_2_; *t* (h)	*x*_1_ *x*_2_	$x_{1}^{2}-2/3$	$x_{2}^{2}-2/3$	*η* (%)
1	–1 (64)	–1 (2.5)	1	1/3	1/3	72
2	–1 (64)	0 (3)	0	1/3	–2/3	76
3	–1 (64)	1 (3.5)	–1	1/3	1/3	70
4	0 (64.5)	–1 (2.5)	0	–2/3	1/3	76
5	0 (64.5)	0 (3)	0	–2/3	–2/3	80
6	0 (64.5)	1 (3.5)	0	–2/3	1/3	77
7	1 (65)	–1 (2.5)	–1	1/3	1/3	75
8	1 (65)	0 (3)	0	1/3	–2/3	76
9	1 (65)	1 (3.5)	1	1/3	1/3	72

**Table 2:** Dimensionless variables and the measured reaction’s yield for obtaining the compound **VI**.

No.	*x*_1_; *T* (°C)	*x*_2_; *t* (h)	*x*_1_ *x*_2_	$x_{1}^{2}-2/3$	$x_{2}^{2}-2/3$	*η* (%)
1	–1 (64)	–1 (3.0)	1	1/3	1/3	80
2	–1 (64)	0 (3.5)	0	1/3	–2/3	83
3	–1 (64)	1 (4.0)	–1	1/3	1/3	78
4	0 (64.5)	–1 (3.0)	0	–2/3	1/3	82
5	0 (64.5)	0 (3.5)	0	–2/3	–2/3	84
6	0 (64.5)	1 (4.0)	0	–2/3	1/3	83
7	1 (65)	–1 (3.0)	–1	1/3	1/3	79
8	1 (65)	0 (3.5)	0	1/3	–2/3	82
9	1 (65)	1 (4.0)	1	1/3	1/3	78

**Table 3:** Dimensionless variables and the measured reaction’s yield for obtaining the compound **VII**.

No.	*x*_1_; *T* (°C)	*x*_2_; *t* (h)	*x*_1_ *x*_2_	$x_{1}^{2}-2/3$	$x_{2}^{2}-2/3$	*η* (%)
1	–1 (64)	–1 (3.5)	1	1/3	1/3	84
2	–1 (64)	0 (4.0)	0	1/3	–2/3	88
3	–1 (64)	1 (4.5)	–1	1/3	1/3	83
4	0 (64.5)	–1 (3.5)	0	–2/3	1/3	86
5	0 (64.5)	0 (4.0)	0	–2/3	–2/3	89
6	0 (64.5)	1 (4.5)	0	–2/3	1/3	85
7	1 (65)	–1 (3.5)	–1	1/3	1/3	84
8	1 (65)	0 (4.0)	0	1/3	–2/3	87
9	1 (65)	1 (4.5)	1	1/3	1/3	83

**Table 4:** The most favorable conditions for obtaining the compounds **V**–**VII**.

Compound	*x*_1_; *T* (°C)	*x*_2_; *t*(h)	*η* (%)
**V**	0.11 (64.55)	–0.09 (3 h 57 min 18 s)	79.83
**VI**	–0.05 (64.47)	0.11 (3 h 33 min 25 s)	84.22
**VII**	–0.05 (64.47)	–0.07 (3 h 57 min 54 s)	89.24

**Table 5:** Tuberculostatic activity of thiosemicarbazides V–VII against *Mycobacterium tuberculosis*, in vitro compared to isoniazid (IAH).

Compound	The Concentration of Thiosemicarbazides in the Culture Medium (μg/mL)										MIC (μg/mL)
	5		10		20		30		40		
	6 Days	15 Days	6 Days	15 Days	6 Days	15 Days	6 Days	15 Days	6 Days	15 Days	
**V**	++	+++	++	++	++	+	–	–	–	–	35
**VI**	++	+++	++	+	–	–	–	–	–	–	20
**VII**	++	+++	+	–	–	–	–	–	–	–	15
**IAH**	–	–	–	–	–	–	–	–	–	–	10

## Data Availability

The data presented in this study are available on request from the corresponding author.
